# Prevalence and correlates of food insecurity among Lebanese households with children aged 4–18 years: findings from a national cross-sectional study

**DOI:** 10.1017/S1368980018003245

**Published:** 2018-12-04

**Authors:** Lamis Jomaa, Farah Naja, Samer Kharroubi, Nahla Hwalla

**Affiliations:** 1 Department of Nutrition and Food Sciences, Faculty of Agricultural and Food Sciences, American University of Beirut, PO Box 11-0.236, Riad El Solh, Beirut, Lebanon; 2 Refugee Health Program, Global Health Institute, American University of Beirut, Beirut, Lebanon

**Keywords:** Food (in)security, Prevalence, Correlates, Households, Lebanon

## Abstract

**Objective:**

Food insecurity (FI) is a major public health problem in Lebanon, a small middle-income country with the highest refugee per capita concentration worldwide and prolonged political and economic challenges. The present study aimed to measure the prevalence and sociodemographic correlates of household FI and to explore the association of household FI with anthropometric measures of children and their mothers.

**Design:**

Cross-sectional survey (2014–2015).

**Setting:**

Lebanon.

**Participants:**

Nationally representative sample of Lebanese households with 4–18-year-old-children and their mothers (*n* 1204).

**Results:**

FI prevalence (95 % CI), measured using the Arabic-translated, validated Household Food Insecurity Access Scale, was found to be 49·3 (44·0, 54·6) % in the study sample. Mild, moderate and severe FI were found in 7·0 (5·5, 9·2) %, 23·3 (20·1, 26·8) % and 18·9 (14·9, 23·5) % of households, respectively. Multiple regression analysis showed that low maternal and paternal education, unemployment and crowding were significant correlates of household FI (*P*<0·05). No significant associations were observed between FI and anthropometric measures of children and their mothers, after adjusting for other socio-economic correlates. Food-insecure households reported various mechanisms to cope with food shortage, such as reducing the number of meals/d (49·6 %), borrowing food (54·4 %), spending savings (34·5 %) and withdrawing children from schools (8·0 %).

**Conclusions:**

FI exists among a remarkable proportion of Lebanese households with children. Correlates of household FI should be considered when designing social welfare policies and public health programmes to promote more sustainable, resilient and healthier livelihoods among vulnerable individuals.

Food insecurity (FI) is defined as the situation where people do not have access, at all times, to sufficient, safe and nutritious food that meets their dietary needs for an active and healthy life while taking into consideration their food preferences^(^ ^1^ ^)^. According to the FAO, one in ten people in the world suffered from severe FI in 2016, corresponding to approximately 688 million people^(^ ^2^ ^)^. Despite international and national efforts to eliminate extreme poverty and increase global availability of food, FI remains a serious problem that affects populations worldwide, particularly those residing in low- and middle-income countries (LMIC)^(^ ^2^ ^)^. Further evidence highlights that FI is concentrated mostly in conflict-affected countries that witness political turmoil, financial instabilities and displacement^(^ ^3^ ^)^.

The Middle East and North Africa (MENA) region is considered one of the most food-insecure regions worldwide, with 11·8 % experiencing severe FI in 2016^(^ ^2^ ^)^. The high prevalence of FI in the MENA region has been attributed to low agricultural production, heavy dependence on food imports, social inequities, economic instabilities and unstable political situations^(^ ^4^ ^)^. In fact, FI and poverty have been argued to be among the main drivers for the ‘Arab Uprisings’ movement that started in 2010, which turned into armed conflicts and protracted crises^(^ ^5^ ^)^. Today, the MENA region has the greatest number of conflicts and refugees worldwide^(^ ^6^ ^)^, with Lebanon, a small middle-income country with a population of just over 6 million people, having the highest per capita concentration of refugees worldwide^(^ ^7^ ^)^. Despite its pertinence, the literature exploring the prevalence and correlates of FI in Lebanon has been mostly focused on refugee populations, including those coming from Syria^(^ ^7^ ^)^, Palestine and Iraq^(^ ^8^ ^,^ ^9^ ^)^, with minimal attention being given to the Lebanese households and communities. The few studies published to date show that FI is not only a rural phenomenon^(^ ^8^ ^)^ but is also experienced among Lebanese urban residents^(^ ^10^ ^,^ ^11^ ^)^.

Exploring the factors that can contribute to household FI in conflict- and displacement-affected settings is challenging, yet it is of utmost importance to identify those most vulnerable to FI. Previous studies show that FI and hunger can be consequences of constrained financial resources; nevertheless, income and poverty measurements alone are not always determinants of FI^(^ ^11^ ^,^ ^12^ ^)^. Other demographic and socio-economic factors can come into play, such as household composition and size, educational attainment, employment status and number of children, including school-aged children^(^ ^12^ ^)^. In addition, households that are vulnerable to poverty and FI may adopt different temporary or permanent coping mechanisms to overcome shortage in economic resources and food^(^ ^13^ ^)^. These mechanisms can vary from dietary restrictions to begging, early marriage and dropping out of school, which can have serious implications on the overall health and well-being of vulnerable household members, particularly women and children^(^ ^14^ ^,^ ^15^ ^)^.

Studies have shown that household FI is associated with inadequate nutrient intakes^(^ ^16^ ^,^ ^17^ ^)^, increased risk of mental health problems and depression^(^ ^18^ ^)^, and poor pregnancy outcomes among women and mothers, including low birth weight and gestational diabetes^(^ ^17^ ^)^. In addition, FI has been associated with maternal thinness in Colombia and Cambodia^(^ ^14^ ^,^ ^19^ ^)^, yet with a higher risk of weight gain and obesity in higher-income countries^(^ ^20^ ^)^. The adverse consequences of FI on children’s health and nutritional status have also been examined, such as increased risk of illnesses, nutrient deficiencies, lower physical and psychological functioning, and increased cognitive and behavioural problems^(^ ^16^ ^,^ ^21^ ^,^ ^22^ ^)^. Nevertheless, these studies have been mostly conducted in high-income countries with few studies exploring the effect of household FI on child health outcomes within LMIC settings^(^ ^14^ ^,^ ^18^ ^,^ ^23^ ^)^. It is also worth noting that these studies focus primarily on young children (<5 years old)^(^ ^19^ ^,^ ^24^ ^)^, with less emphasis being placed on older children^(^ ^25^ ^)^.

Recent evidence highlights the importance of considering school-aged children, who may be equally, if not even more vulnerable to the adverse consequences of household FI compared with their younger siblings^(^ ^25^ ^–^ ^27^ ^)^. Older children may be at increased risk of health insults associated with FI due to their dependence on the table food available in the household compared with their younger siblings, who may be still protected nutritionally by breast-feeding and other early infant feeding practices^(^ ^25^ ^)^. In addition, school-aged children have higher nutritional needs, yet mothers may be less attentive to their dietary intakes or may have less control over their older children’s choice of foods and beverages outside the home setting compared with the younger ones^(^ ^27^ ^)^.

To address this gap in the literature, the present study aimed to: (i) estimate the prevalence of FI using a nationally representative sample of Lebanese households with children aged 4–18 years and their mothers; (ii) examine the sociodemographic correlates of household FI in the study sample; (iii) explore the associations between household FI and anthropometric measurements of children and their mothers; and (iv) identify the coping mechanisms adopted by food-insecure households.

## Materials and methods

### Study population

Data for the present study were drawn from a cross-sectional survey, the Lebanese Food and Nutrition Security Survey (L-FANUS), conducted on a nationally representative sample of Lebanese households with children aged 4–18 years. Data collection took place between December 2014 and November 2015, which overlapped with the fifth year of the Syrian crisis. A stratified cluster sampling strategy was followed, whereby the strata were the six Lebanese governorates and the clusters were drawn from the twenty-six districts (*Caza*) in Lebanon. The number of clusters per district differed depending on the population density in that district. In each district, households were selected based on a probability-proportional-to-size approach. A higher number of participating households was drawn from districts with higher population density using data from the Lebanese Central Administration of Statistics^(^ ^28^ ^)^ and after consulting with a local statistics agency. Households were selected using a systematic sampling approach. Sample size calculations showed that a minimum of 1200 households was needed to provide 95 % CI for a FI prevalence estimate of 51·7 % with ±4 % variation and a design effect of 2. The estimate of 51·7 % used in the sample size calculation was based on the FI estimate reported among a small sample of households in Lebanon^(^ ^29^ ^)^.

For a household to be eligible, it had to include a mother and one child between 4 and 18 years of age who were present at the time of the interview. In cases where more than one child was eligible, only one was selected at random to participate in the study using the Kish method^(^ ^30^ ^)^. Inclusion criteria were having the mother and child be of Lebanese nationality, not suffering from any chronic disease, and not taking any medications that may interfere with their body composition or their overall nutritional status. Of the 4076 households that were contacted, 3147 accepted to participate in the study (response rate=77 %) and 1221 households met the eligibility criteria. Of these, 1209 completed the interview. The main reasons for refusing to participate in the study were time constraints and disinterest in the study. Informed consent and assent were obtained from mothers and their children prior to their participation in the study.

### Data collection

Face-to-face interviews with study participants were held in the household setting and lasted on average 45 min. Mothers served as the main proxy for the household. The survey included sociodemographic questions such as age of mother and child, child’s gender, number of children in the household, household income, educational level and employment status of parents, and region of residence (governorate). In addition, the total number of persons and rooms per household were collected to calculate crowding index. The latter was calculated as the total number of household members divided by the total number of rooms in a household (excluding kitchens, bathrooms and balconies)^(^ ^31^ ^)^. Households with fewer than two persons per room were considered to have a lower crowding index as compared with those with two or more persons per room. This index was used as a proxy measure of household socio-economic status in the present study, as it was previously used in Lebanon and provided reliable results^(^ ^29^ ^,^ ^31^ ^,^ ^32^ ^)^.

To measure household FI, the survey included nine questions from the Household Food Insecurity Access Scale (HFIAS). The HFIAS was originally developed by the US Agency for International Development-funded Food and Nutrition Technical Assistance Project^(^ ^33^ ^)^. This tool was previously translated to Arabic and validated in Lebanon, showing high internal consistency (Cronbach’s *α* of 0·91) and reliability (intraclass correlation coefficient of 0·58)^(^ ^29^ ^)^. This nine-question scale produces a total score between 0 and 27 with higher scores indicating greater FI; results from this scoring are used to categorize households into four levels of FI (food secure, mildly, moderately or severely food-insecure households) depending on the number of positive responses to questions related to severe conditions and as per the HFIAS measurement and indicator guide^(^ ^33^ ^)^.

The coping mechanisms of households in the study sample were assessed using an adapted version of the Coping Strategies Index (CSI). Households who reported that they had shortage in money and food in the past month completed this section of the survey. The adapted CSI tool included two sets of questions about the coping strategies adopted by households: (i) food-related strategies such as relying on less preferred and less expensive foods, borrowing food, limiting portion sizes at meal times and reducing number of meals eaten in a day; and (ii) asset-related strategies such as spending savings, selling jewellery or household goods (furniture, television, radio, etc.), selling assets (house, land, transportation mode), reducing essential non-food expenditures on health and education, withdrawing children from school and having children (6–15 years old) be involved in income-generating activities.

### Anthropometric measurements of mothers and children

Anthropometric assessments for study participants were conducted by trained dietitians using standard techniques and equipment. Participants were weighed on a digital scale (Seca model 770, Hamburg, Germany) to the nearest 0·1 kg while wearing light clothing. Height was measured using a portable stadiometer (Seca model 213, Hamburg, Germany) to the nearest 0·1 cm while not wearing shoes and in a standing position. Waist circumference was measured to the nearest 0·1 cm using a plastic measuring tape at the midpoint between the bottom of the rib cage and the top of the iliac crest. All measurements were taken twice and the average of the two values was reported. BMI was calculated by dividing weight by height squared (kg/m^2^). Mothers were categorized as normal weight, overweight or obese based on the WHO classifications^(^ ^34^ ^)^. BMI-for-age *Z*-scores (BAZ) for children and adolescents were calculated using the WHO Anthro Plus software version 1.0.4^(^ ^35^ ^)^. Children were classified as thin, normal weight, at risk of overweight (only for children<5 years), overweight and obese based on the WHO age-specific cut-offs^(^ ^36^ ^)^. Height-for-age *Z*-score (HAZ) and weight-for-age *Z*-score (WAZ) were calculated to assess undernutrition among children in the study sample. Stunting and underweight were defined as HAZ<–2 and WAZ<–2 compared with the WHO child growth standards, respectively^(^ ^35^ ^)^. In addition, waist-to-height ratio (WHtR) was calculated as waist circumference divided by height, both being measured in centimetres. The suggested cut-off point of WHtR≥0·5 was used to identify children with elevated WHtR, an indicator of abdominal obesity among children^(^ ^37^ ^)^.

### Statistical analyses

Descriptive statistics were reported as means with their standard errors for continuous variables and as frequencies and proportions for categorical variables. As noted earlier, households were recruited from the six Lebanese governorates, namely Beirut (the capital), Bekaa, Mount Lebanon, North of Lebanon, Nabatiyeh and South of Lebanon. The two governorates located in the southern regions of the country (Nabatiyeh and South of Lebanon) were grouped together for analysis. The results in the present study were weighted to account for the effect of the cluster sampling technique used in L-FANUS. The weighted analyses were conducted using the statistical software package Stata/SE version 12. The prevalence of FI was reported at the national and governorate levels. The associations of sociodemographic and anthropometric characteristics of the study sample with the odds of household FI were conducted using simple and multiple logistic regressions. Anthropometric variables explored in the logistic regression models included BMI for mothers, and BAZ, WAZ, HAZ and WHtR for children. The outcome in these models was household FI, which was recoded into two categories: 0=food secure *v*. 1=food insecure (combined mildly, moderately and severely food-insecure households). All sociodemographic variables that were found significant in the simple logistic models were entered in the final multiple logistic regressions. Results from the logistic regression models were expressed as odds ratios with 95 % confidence intervals. *P*<0·05 was considered statistically significant.

## Results

Out of the 1209 households with children aged 4–18 years who took part in the study, only five households had incomplete sociodemographic and food security data and were thus excluded from the analyses. Table 1 shows the sociodemographic characteristics of the weighted study sample. On average, mother’s age was 39·6 (se 0·3) years and children were 10·9 (se 0·2) years old, with an average of three children per household. The sample included an equal distribution of boys and girls (48·4 and 51·6 %, respectively). The educational levels of parents were found to be comparable, with 55·3 % of households having fathers with intermediate school level education or less and 47·4 % having mothers with a similar educational attainment. The majority of households had employed fathers (94 %), whereas about 24 % of households had employed mothers. In addition, 26 % had a crowding index of ≥2 persons/room and 40 % of households had monthly income less than $US 663, a value slightly higher than the minimum wage salary in Lebanon of $US 450.

**Table 1:** Sociodemographic and anthropometric characteristics of the study sample of Lebanese households with children aged 4–18 years (*n* 1204), 2014–2015†

	Mean or *n*	se or %
Sociodemographic characteristics		
Child’s age (years)	10·9	0·18
Mother’s age (years)	39·6	0·32
Number of children	3·1	0·08
Child’s gender		
Male	582	48·4
Female	621	51·6
Father’s education level		
Intermediate school or less	658	55·3
High school/technical diploma	367	30·8
University degree	165	13·9
Mother’s education level		
Intermediate school or less	571	47·4
High school/technical diploma	400	33·2
University degree	233	19·4
Father’s employment status		
Unemployed	69	5·9
Employed	1109	94·1
Mother’s employment status		
Unemployed	916	76·3
Employed	285	23·7
Crowding index		
<2 persons/room	894	74·4
≥2 persons/room	308	25·6
Household monthly income (Lebanese pounds)		
<1 000 000 (equivalent to<$US 663)	470	39·8
1 000 000–2 000 000 ($US 663–1326)	441	37·3
>2 000 000 (equivalent to >$US 1326)	270	22·9
Region of residence (governorate)		
Beirut	62	5·1
Bekaa	215	18·1
Mount Lebanon	468	38·9
North of Lebanon	198	16·0
South of Lebanon	261	21·9
Anthropometric measures‡		
Mother’s BMI		
Normal weight (18·5–24·9 kg/m^2^)§	378	31·5
Overweight (25·0–29·9 kg/m^2^)	422	35·2
Obese (≥30·0 kg/m^2^)	399	33·3
Children (4–5 years)		
BMI status		
Thin (BAZ<−2)	0	0·0
Normal (−2≤BAZ≤+1)	45	58·2
At risk of overweight (+1<BAZ≤+2)	9	11·5
Overweight (+2<BAZ≤+3)	16	20·0
Obese (BAZ>+3)	8	10·6
Children (>5 years)		
BMI status		
Thin (BAZ<−2)	16	1·5
Normal weight (−2≤BAZ≤+1)	617	55·8
Overweight (+1<BAZ≤+2)	249	22·5
Obese (BAZ>+2)	223	20·2
WHtR≥0·5 (elevated)║	347	31·5
HAZ<−2 (stunted)	57	4·8
WAZ<−2 (underweight)¶	12	2·4

BAZ, BMI-for-age *Z*-score; WHtR, waist-to-height ratio; HAZ, height-for-age *Z*-score; WAZ, weight-for-age *Z*-score.

† Continuous variables are presented as means with their se, whereas categorical variables are reported as *n* and %.

‡ Anthropometric measurements of mothers and children were categorized based on WHO classification^(^ ^34^ ^–^ ^36^ ^)^.

§ Underweight mothers (*n* 11) were added to the normal-weight group.

║ WHtR was calculated only for children aged >5 years.

¶ WAZ was calculated only for children aged<10 years as per the WHO growth charts.

In terms of the anthropometric characteristics of the study sample, more than two-thirds of mothers were either overweight or obese (68·5 %) with the remaining 31·5 % having normal body weight. For children (4–5 years), 58·2 % were found to be of normal weight while the remaining children were classified as at risk of overweight (11·5 %), overweight (20·0 %) or obese (10·6 %). As for children above 5 years of age, approximately 56 % were normal weight while 22 % were overweight and 20 % were obese. In addition, slightly less than a third of children in the study sample had an elevated WHtR (WHtR≥0·5) and less than 5 % of children were found to be either stunted or underweight using the age- and gender-specific growth charts.

The prevalence of FI reported in the present study sample was 49·3 % (95 % CI 44·0, 54·6 %). Approximately 7 % of households (95 % CI 5·5, 9·2 %) were mildly food insecure, 23·3 % (95 % CI 20·1, 26·8 %) were moderately food insecure and 18·9 % (95 % CI 14·9, 23·5 %) were severely food insecure. Using simple logistic regression analysis, the correlates of FI were mother’s age, number of children, mother and father’s educational attainment and employment status, region of residence, crowding index and income (Table 2). Results from the multiple logistic regression model showed that households with employed fathers and employed mothers were significantly less likely to be food insecure than those with unemployed fathers and mothers (OR=0·35; 95 % CI 0·17, 0·73 and OR=0·54; 95 % CI 0·35, 0·84, respectively). In addition, households with higher parental educational levels were significantly less likely to be food insecure than households with lower parental educational levels (*P*<0·05; Table 2). Households with higher crowding (≥2 persons/room) remained significantly associated with higher odds of household FI (OR=1·86; 95 % CI 1·26, 2·75), even after adjusting for other socio-economic correlates. Household income was excluded from the multiple logistic regression models to avoid multicollinearity with crowding index. Data from previous studies also showed that crowding index is less subject to reporting bias than income, and it can be a more reliable measure of a household’s socio-economic status^(^ ^38^ ^)^.

**Table 2:** Associations of sociodemographic characteristics with the odds of household food insecurity in the study sample of Lebanese households with children aged 4–18 years (*n* 1204), 2014–2015†

Sociodemographic characteristic	Food secure (*n* 569)		Mildly food secure (*n* 100)		Moderately food insecure (*n* 274)		Severely food insecure (*n* 261)		OR‡	95 % CI	Adjusted OR§	95 % CI
	Mean or *n*	se or %	Mean or *n*	se or %	Mean or *n*	se or %	Mean or *n*	se or %				
Child’s age (years)	10·7	0·29	10·8	0·52	11·0	0·31	11·7	0·29	1·04	0·99, 1·08	–	–
Mother’s age (years)	39·0	0·48	39·5	0·78	40·1	0·58	40·6	0·57	1·02^*^	1·00, 1·04	1·00	0·98, 1·03
Number of children	2·8	0·06	2·8	0·11	3·3	0·12	3·6	0·22	1·38^**^	1·21, 1·56	1·10	0·97, 1·26
Child’s gender												
Male	278	48·9	50	50·1	120	43·7	137	52·4	1·00	Ref.	–	–
Female	290	51·1	50	49·9	154	56·3	124	47·6	1·04	0·78, 1·39	–	–
Father’s education level												
Intermediate school or less	230	40·5	58	58·1	189	69·7	194	76·5	1·00	Ref.	1·00	Ref.
High school/technical diploma	218	38·6	30	30·5	65	24·2	45	17·9	0·34^**^	0·23, 0·49	0·58^**^	0·39, 0·87
University degree	118	20·9	11	11·4	17	6·1	15	5·6	0·19^**^	0·11, 0·32	0·43^**^	0·23, 0·79
Mother’s education level												
Intermediate school or less	160	28·0	52	51·7	181	66·0	195	74·8	1·00	Ref.	1·00	Ref.
High school/technical diploma	246	43·3	32	31·8	59	21·4	56	21·3	0·22^**^	0·16, 0·31	0·31^**^	0·21, 0·47
University degree	163	28·7	16	16·5	34	12·6	10	3·9	0·14^**^	0·09, 0·22	0·30^**^	0·18, 0·50
Father’s employment status												
Unemployed	14	2·5	7	7·1	14	5·3	39	15·7	1·00	Ref.	1·00	Ref.
Employed	547	97·5	91	92·9	256	94·7	210	84·3	0·24^**^	0·11, 0·51	0·35^**^	0·17, 0·73
Mother’s employment status												
Unemployed	382	67·3	83	83·6	228	83·1	233	89·3	1·00	Ref.	1·00	Ref.
Employed	185	32·7	16	16·4	46	16·9	28	10·7	0·35^**^	0·24, 0·50	0·54^**^	0·35, 0·84
Crowding index												
<2 persons/room	485	85·6	85	85·2	178	65·2	135	51·9	1·00	Ref.	1·00	Ref.
≥2 persons/room	82	14·4	15	14·8	96	34·8	126	48·1	3·48^**^	2·41, 5·03	1·86^**^	1·26, 2·75
Region of residence (governorate)												
Beirut	32	5·6	5	4·1	8	3·0	17	6·6	1·00	Ref.	1·00	Ref.
Bekaa	114	20·0	15	14·9	58	21·1	28	10·6	1·00	0·52, 1·93	0·73	0·38, 1·41
Mount Lebanon	225	39·6	50	50·2	100	36·5	93	35·7	1·20	0·72, 2·00	1·40	0·83, 2·51
North of Lebanon	65	11·4	11	11·4	45	16·3	77	29·6	2·30^*^	1·08, 4·73	1·30	0·72, 2·29
South of Lebanon	133	23·4	19	19·4	63	23·1	46	17·5	1·10	0·59, 1·99	0·72	0·37, 1·42
Household monthly income (Lebanese pounds)║												
<1 000 000	105	18·9	45	46·8	159	58·9	177	68·5	1·00	Ref.	–	–
1 000 000–2 000 000	232	41·7	38	39·0	98	36·4	68	26·2	0·25^**^	0·16, 0·37	–	–
>2 000 000	219	39·3	14	14·2	13	4·7	13	5·2	0·05^**^	0·03, 0·09	–	–

Ref., reference category.

* *P*<0·05

** *P*<0·01.

† Continuous variables are presented as means with their se, whereas categorical variables are reported as *n* and %.

‡ OR of the dependent variable (food-insecure *v*. food-secure households) are presented with 95 % CI using simple logistic regression. The food-insecure category included mildly, moderately and severely food-insecure households.

§ Adjusted OR are presented with 95 % CI using multiple logistic regression analysis. The models were adjusted for sociodemographic characteristics found to be significant correlates of food insecurity (mother’s age, number of children, parental education and employment status, region of residence and crowding index), except for income to avoid multicollinearity.

║ Household income was excluded from the multiple logistic regressions to avoid multicollinearity with crowding index.

Table 3 presents the associations between household FI status and the anthropometric measures of mothers and children. Using simple logistic regression analysis, FI was found to be 1·79 times more likely among households in which the mothers were obese as compared with households in which the mothers had normal weight. However, this association was no longer statistically significant after adjusting for other sociodemographic correlates of FI. No other significant associations were observed between household FI and anthropometric measurements of children in the multiple logistic regression models.

**Table 3:** Associations of anthropometric measures with the odds of household food insecurity in the study sample of Lebanese households with children aged 4–18 years (*n* 1204), 2014–2015†

Anthropometric measure†	Food secure (*n* 569)		Mildly food insecure (*n* 100)		Moderately food insecure (*n* 274)		Severely food insecure (*n* 261)		OR‡	95 % CI	Adjusted OR§	95 % CI
	*n*	%	*n*	%	*n*	%	*n*	%				
Mother’s BMI												
Normal weight (18·5–24·9 kg/m^2^)║	200	35·3	35	35·5	68	25·1	72	27·7	1·00	Ref.	1·00	Ref.
Overweight (25·0–29·9 kg/m^2^)	209	36·9	37	37·0	93	34·1	82	31·5	1·16	0·79, 1·71	1·01	0·67, 1·52
Obese (≥30·0 kg/m^2^)	157	27·8	28	27·6	112	40·8	106	40·7	1·79^**^	1·16, 2·76	1·39	0·84, 2·29
Children (4–5 years)												
BMI status												
Normal (−2≤BAZ≤+1)	26	62·0	3	29·2	2	72·9	3	47·2	1·00	Ref.	1·00	Ref.
At risk of overweight/overweight (BAZ>+1)	16	38·0	8	70·8	2	27·1	6	52·8	1·28	0·75, 2·18	1·38	0·64, 2·94
Children (>5 years)												
BMI status												
Normal weight (BAZ≤+1)	300	58·2	51	57·1	142	55·4	140	57·5	1·00	Ref.	1·00	Ref.
Overweight (+1<BAZ≤+2)	116	22·5	23	25·9	59	23·1	50	20·7	1·03	0·65, 1·64	1·05	0·61, 1·79
Obese (BAZ>+2)	100	19·3	15	17·0	56	21·5	53	21·8	1·12	0·76, 1·64	1·31	0·84, 2·03
WHtR¶												
WHtR<0·5	367	71·2	64	72·8	168	65·0	155	63·9	1·00	Ref.	1·00	Ref.
WHtR≥0·5 (elevated)	148	28·8	24	27·2	90	35·0	87	36·1	1·29	0·95, 1·76	1·23	0·83, 1·83
HAZ												
HAZ<−2 (stunted)	30	5·3	2	1·9	5	1·9	21	8·0	1·00	Ref.	1·00	Ref.
HAZ≥−2	534	94·7	98	98·1	263	98·1	238	92·0	1·27	0·06, 2·78	1·61	0·70, 3·87
WAZ††												
WAZ<−2 (underweight)	7	2·7	2	4·6	0	0·0	3	3·6	1·00	Ref.	1·00	Ref.
WAZ≥−2	249	92·3	39	95·4	109	100·0	91	96·4	1·42	0·25, 7·97	1·59	0·38, 6·61

BAZ, BMI-for-age *Z*-score; WHtR, waist-to-height ratio; HAZ, height-for-age *Z*-score; WAZ, weight-for-age *Z*-score; Ref., reference category.

* *P*<0·05

** *P*<0·01.

† Anthropometric measurements of mothers and children were categorized based on WHO classification^(^ ^34^ ^–^ ^36^ ^)^.

‡ OR of the dependent variable (food-insecure *v.* food-secure households) are presented with 95 % CI using simple logistic regression. The food-insecure category included mildly, moderately and severely food-insecure households.

§ Adjusted OR are presented with 95 % CI using multiple logistic regression analysis. The models were adjusted for sociodemographic characteristics found to be significant correlates of food insecurity (mother’s age, number of children, parental education and employment status, region of residence and crowding index), except for income to avoid multicollinearity.

║ Underweight mothers (*n* 11) were added to the normal-weight group.

¶ WHtR was calculated for children aged>5 years only.

†† WAZ was calculated only for children aged<10 years as per the WHO growth charts.

Table 4 presents coping mechanisms of food-insecure households in the study sample who reported that they did not have enough food or money to buy food for their family members during the past month. The most commonly adopted coping strategies reported by food-insecure households (whether sometimes, often or always in the past month) included food-related strategies such as relying on less preferred and less expensive foods (81·2 % of households), borrowing food or relying on help from a friend or relative to secure food (54·4 %), reducing the number of meals eaten per day (49·6 %) and restricting the consumption of adults to secure food for children (45·5 %). Food-insecure households also resorted to various asset-related strategies to cope with their condition, from spending savings, selling assets (jewellery, household goods, house/land) and reducing essential non-food expenditures on education and health, to more severe coping mechanisms such as withdrawing children from school (8·0 %) and involving them in income-generating activities (8·7 %).

**Table 4:** Coping mechanisms adopted by food-insecure households who reported that they did not have enough food or money to buy food for their family members in the past month (*n* 506) in the study sample of Lebanese households with children aged 4–18 years, 2014–2015

Coping strategy	Always		Often		Sometimes		Never	
	*n*	%	*n*	%	*n*	%	*n*	%
Food-related strategies								
Relied on less preferred and less expensive foods	150	29·8	125	24·8	134	26·6	95	18·8
Borrowed food or relied on help from a friend/relative	101	20·0	66	13·1	107	21·2	230	45·6
Limited portion size at meal times	31	6·2	50	9·9	89	17·7	333	66·2
Restricted consumption by adults for small children to eat	30	3·0	61	12·1	138	27·4	274	54·5
Reduced number of meals eaten in a day	41	8·1	74	14·7	135	26·8	254	50·4
Sent family members eat elsewhere	23	4·6	29	5·8	66	13·2	382	76·4
Borrowed money to buy food	21	4·2	45	9·0	49	9·8	387	77·1
Asset-related and other coping strategies								
Spent savings	70	13·9	37	7·4	66	13·1	329	65·5
Sold jewellery or household goods (furniture, television, radio, etc.)	43	8·5	22	4·3	55	10·9	386	76·3
Sold assets: house or land	15	3·0	5	1·0	8	1·6	476	94·4
Sold assets: transportation mode	3	0·6	2	0·4	6	1·2	493	97·8
Reduced essential non-food expenditures such as education, health	55	11·0	33	6·6	77	15·3	337	67·1
Withdrew children from school	26	5·2	7	1·4	7	1·4	463	92·0
Involved schoolchildren (6–15 years old) in income generation	24	4·8	7	1·4	13	2·6	461	91·3

## Discussion

The present study is the first to explore the prevalence and correlates of FI among a nationally representative sample of Lebanese households with children aged 4–18 years. Findings show that almost half of the study sample was food insecure in 2015, with more than 42·2 % being moderately to severely food insecure. The overall FI prevalence in the present study was found to be comparable to those reported in previous studies conducted among smaller samples of Lebanese households in the South of Lebanon and Greater Beirut regions (42 and 50 %, respectively)^(^ ^10^ ^,^ ^11^ ^)^. Nevertheless, FI prevalence in our study sample remained higher than those reported in other middle-income countries in the MENA region, including Jordan, Turkey and Egypt (17–32 %)^(^ ^39^ ^–^ ^41^ ^)^; and it was much higher than prevalence rates reported in high-income countries, such as the USA, Canada and France, with only 5–15 % of their respective populations experiencing FI^(^ ^12^ ^,^ ^42^ ^,^ ^43^ ^)^.

The high prevalence of FI in the study sample needs to be considered in the light of the country’s historical and current conditions. Lebanon has undergone persistent conflicts and volatile political and economic conditions for more than three decades, and since 2011 it has been further challenged with the influx of large numbers of Syrian refugees into the country. The prolonged displacement of Syrian refugees has added major strain on the Lebanese economy and infrastructure and may have jeopardized the food security of its population^(^ ^44^ ^)^. Data from the 2007 national report on poverty, growth and income distribution in Lebanon showed that approximately 30 % of the population lived below the poverty line, and 8 % of the Lebanese population continued to live in extreme poverty (below $US 2·40/person per d^(^ ^45^ ^,^ ^46^ ^)^). More recent data from the World Bank showed that the number of people living under the poverty line in Lebanon has risen by 66 % since the start of the Syrian refugee crisis, pushing almost 170 000 Lebanese into poverty and doubling unemployment to over 20 %, especially among the unskilled youth^(^ ^44^ ^,^ ^46^ ^)^. In parallel, 1·3 million people were identified to be in need of food post the crisis, of which 182 000 were from the most vulnerable Lebanese communities hosting Syrian refugees and displaced families^(^ ^47^ ^)^. All these conditions and others may have contributed to the economic challenges and FI status of the most vulnerable households in the country, particularly those with children.

When exploring correlates of FI, findings from the present study showed that household FI was associated with several sociodemographic characteristics, including lower parental educational attainment and unemployment together with higher household crowding. These results were in line with a study conducted in Lebanon on a smaller sample of households with young children (<2 years) showing significant associations between HFIAS scores and monthly income (*r*=−0·45), crowding index (*r*=0·3), and mother’s and father’s educational levels (*r*=−0·47 and −0·22, respectively)^(^ ^8^ ^)^. Similarly, lower odds of FI were previously associated with higher maternal education and household incomes among Lebanese households in the Greater Beirut region^(^ ^9^ ^)^. Evidence from other middle- and higher-income countries corroborates our findings showing that low educational attainment, unemployment, household composition and size, and having more children, including school-going children, were strong correlates of FI^(^ ^12^ ^,^ ^23^ ^,^ ^48^ ^)^.

Findings from the present study show that food-insecure households resorted to various food- and asset-based coping mechanisms, such as reducing the number and portion size of meals consumed per day, borrowing food or money to buy food, spending savings, and reducing essential non-food expenditures such as on education and health. Similar coping strategies were previously reported among Syrian, Iraqi and Palestinian refugees in Lebanon^(^ ^7^ ^–^ ^9^ ^)^, such as borrowing money, accepting a gift or donation, and consuming cheaper food items. The adoption of such mechanisms among households with children is particularly alarming, as it can have serious detrimental effects on the physical, cognitive and psychological health of children in the short and long run, and it warrants further attention^(^ ^18^ ^,^ ^22^ ^,^ ^25^ ^)^.

The associations between household FI and the anthropometric measures of mothers and children were also explored in the present study. Although FI was 1·79 times more common among households in which the mothers were obese compared with households with normal-weight mothers, this association was no longer statistically significant after adjusting for other socio-economic covariates. These results were in contrast to those reported earlier in Beirut showing a significant association between household FI and maternal obesity (OR=1·73; 95 % CI 1·02, 2·92). Given that household FI was found to be significantly associated with maternal education and employment status in the present study, adjusting for these variables in the regression models may have attenuated the association between FI and obesity. According to a recent study conducted using national health surveys from thirty-eight LMIC, the formal employment of mothers was found to be significantly associated with higher odds of overweight, yet the magnitude of the association was affected by the educational attainment of mothers and not the countries’ gross domestic product or urbanization^(^ ^49^ ^)^. These results may explain why the relationship between FI and maternal obesity is less consistent in LMIC^(^ ^39^ ^,^ ^49^ ^)^ compared with the strong evidence from high-income countries^(^ ^16^ ^,^ ^50^ ^)^.

A high prevalence of overweight and obesity among 4–18-year-old children was observed in the present study (22·5 and 20·2 %, respectively), yet no significant associations were observed between household FI and childhood obesity. A previous national study conducted in Lebanon showed a similar high prevalence of overweight and obesity among 6–19-year-old children (34·8 and 13·2 %, respectively). In addition, maternal education and employment status were associated with higher odds of overweight and obesity, respectively, among 6–11-year-old children, but not among older children (12–19 years old)^(^ ^32^ ^)^. The scientific literature shows mixed evidence with respect to the relationship between FI and obesity among children and adolescents from various contexts^(^ ^50^ ^,^ ^51^ ^)^. Thus, it is critical to explore the underlying mechanisms that can help explain the FI–obesity paradox and devise evidence-based strategies to curb the rising rates of paediatric overweight and obesity, particularly in LMIC settings.

The current study has a number of strengths. It is the first to explore the prevalence and correlates of household FI among a representative sample of Lebanese households with children. The demographic and socio-economic characteristics of the study sample were found to be comparable to national figures most recently available in Lebanon^(^ ^47^ ^,^ ^51^ ^)^. Other strengths of the study include the use of a culturally sensitive questionnaire, a locally validated household food security access scale, and the collection of anthropometric measurements by trained dietitians to increase the accuracy of data and reduce the risk of reporting bias. However, results from the present study need to be interpreted in the light of a number of limitations. The study sample is not representative of the Lebanese households in general, but rather of households that have children aged 4–18 years. Another limitation is the inability to report household income per capita in the present study given that income was collected as a categorical rather than a numerical variable. Future studies need to consider income per capita and to explore other socio-economic and neighbourhood characteristics when exploring correlates of household FI. The cross-sectional design of the study allowed us only to examine associations rather than explore potential causal pathways between FI and various sociodemographic characteristics and maternal and childhood nutritional status. FI was also measured at the household level and it may not reflect the severity of FI that is witnessed at the individual level. Nevertheless, every attempt was made to ensure that mothers, as proxy respondents, considered the food security of all household members when responding to these questions.

## Conclusion

Findings from the present study show that the prevalence of FI was notably high among Lebanese households with children. Correlates of FI included crowding of the household, low parental educational attainment and unemployment. Although no associations were found between household FI and children’s and mother’s anthropometric measures after adjusting for sociodemographic correlates, both groups require particular attention. FI increases the risk for women and children to adopt unhealthy eating and lifestyle behaviours, which can have long-lasting effects on their overall health and well-being. Findings from the present study highlight the need for identifying households most vulnerable to FI and for devising evidence-based policies and programmes that can help promote more sustainable, resilient and healthier livelihoods.
